# Thyroid Disrupting Chemicals

**DOI:** 10.3390/ijms18122583

**Published:** 2017-12-01

**Authors:** Valeria Calsolaro, Giuseppe Pasqualetti, Filippo Niccolai, Nadia Caraccio, Fabio Monzani

**Affiliations:** 1 Department of Clinical & Experimental Medicine, University of Pisa, Pisa 56125, Italy; valina82@gmail.com (V.C.); giuseppe.pasqualetti@gmail.com (G.P.); filippo.niccolai@alice.it (F.N.); nadiacaraccio.amb@libero.it (N.C.); 2 Neurology Imaging Unit, Imperial College, London W12 0NN, UK

**Keywords:** thyroid, thyroid hormone, disrupting compound, hypothalamus pituitary thyroid axis, Thyroid-Stimulating Hormone (TSH)

## Abstract

Endocrine disruptor compounds are exogenous agents able to interfere with a gland function, exerting their action across different functional passages, from the synthesis to the metabolism and binding to receptors of the hormone produced. Several issues, such as different levels and time of exposure and different action across different ages as well as gender, make the study of endocrine disruptors still a challenge. The thyroid is very sensitive to the action of disruptors, and considering the importance of a correct thyroid function for physical and cognitive functioning, addressing this topic should be considered a priority. In this review, we examined the most recent studies, many of them concentrating on maternal and child exposure, conducted to assess the impact of industrial chemicals which showed an influence on thyroid function. So far, the number of studies conducted on that topic is not sufficient to provide solid conclusions and lead to homogeneous guidelines. The lack of uniformity is certainly due to differences in areas and populations examined, the different conditions of exposures and the remarkable inter-subject variability. Nonetheless, the European Commission for Health and Food Safety is implementing recommendations to ensure that substances identified as endocrine disruptors will be withdrawn from the market.

## 1. Introduction

According to the US Environmental Protection Agency (EPA) an endocrine disrupting compound (EDC) is defined “an exogenous agent that interferes with synthesis, secretion, transport, metabolism, binding action or elimination of natural blood-borne hormones that are present in the body and are responsible for homeostasis, reproduction, and developmental process” [1]. While few years ago the action of EDCs was thought to be exerted mainly through nuclear hormone receptors, it is now widely accepted that membrane, orphan and neurotransmitters receptors, as well as several enzymatic pathways, are involved and impaired in the endocrine disruptive process [1]. Some studies have shown that chemical substances considered individually do not have negative effects on the organism (NOEL, No Observed Effect Level), while they have if more substances are evaluated simultaneously. Therefore, this shifts the attention toward mixtures of compounds which are potentially harmful. In fact, a subsequent scientific statement from the Endocrine Society defined the EDC as “an exogenous chemical, or mixture of chemicals, that interferes with any aspect of hormone action” [2]. Mixtures in particular constitute a complex problem; in the late 70s, the production of Polychlorinated Biphenyls (PCB) in the US was banned by the Congress, because commercial mixture of PCB showed a dioxine-like effect [2]. The studies conducted on the matter are mainly concentrated to the exposition to single compounds, or more compounds but separately analyzed, even if the environmental exposure is mainly to a mixture of compounds [3]. A recent animal study showed that there is a different response in hormonal status and gene expression between the exposure to a mixture compared to the exposure to a single compound [3]. What makes the EDCs action on the endocrine system so difficult to evaluate is the broad spectrum of different chemicals potentially involved, the different age of exposure and duration of the exposure itself, the difference in the decay of the compounds, the variable contamination of water/soil and the geographical differences [1]. The difference in exposition time points during lifetime is also a critical factor in the evaluation of EDC actions. The age of exposure is the first conditioning factor, implying the maternal exposure as well; moreover, the variable latency between the exposure and the manifestation of the effect needs to be taken in to consideration. In addition to these factors, it is important to bear in mind that the action of an EDC might be potentiated or somehow modified by the mixture with different other polluters in the environment [4]. Moreover, the dose-effect relationship is not predictable and not necessarily linear; low doses of endocrine disruptors could determine effects not consistent with the ones seen at highest doses [4]. More specifically, low-dose exposure and nonmonotonic dose-response curves are major issue to be taken into consideration when evaluating EDCs. Low dose effect has been variously defined, considering that while endogenous hormones exert effect even at picomolar concentration, environmental ones are active at greater ranges (nano or micromolar) [4]. The concept is rather an operational definition, considering “low doses” the ones in the range of human exposures or below the threshold used in toxicological studies [4]. Great importance has the nonmonotonic dose-response curves. A nonmonotonic dose-response is a curve response with a slope that can change from a U-shape to an inverted U-shape or viceversa within the range of the doses tested, resulting in a challenge when approaching the study of environmental disruptors action on endocrine system [4,5]. Another mechanism suggested is the potential indirect action on regulation of gene expression, dealing with potential inheritable effects [1]. Thyroid tissue is very sensitive to EDC and, considering the impact that the thyroid has in the physical development, the cognitive and neuronal functioning as well as the intermediate metabolism [6], a negative impact on thyroid function might have important repercussions on neuronal and physical health and development [1,7]. It is important to notice that, due to the peculiarity of the thyroid tissue functioning, iodine deprivation might be a predisposing condition to the adverse effects of ECDs [7]. Whatever the mechanism involved, the detrimental effect of some chemicals on the endocrine system and population health is widely recognized not negligible [7]. Accordingly, on July 2017 EU member States representatives voted in favour of the European Commission’s proposal on scientific criteria to identify endocrine disruptors in the field of plant protection products, considering it an important step towards greater protection of citizens from harmful substances [8]. The criteria endorsed by the European Commission concerning substances falling within the plant protection products legislation are based on the World Health Organization (WHO) definition. They identify known and presumed endocrine disruptors. They also specify that the identification of an endocrine disruptor should be carried out by taking into account all relevant scientific evidence including animal, in vitro or in silico studies, and using a weight of evidence-based approach [8]. Giving these premises the current review aimed at evaluating the different endocrine disrupting compounds and their action on thyroid function as assessed either in humans or animals as well as in in vitro studies. To do so, we reviewed the English scientific literature available on National Library of Medicine (www.pubmed.com) since 1994, with particular focus on the more recent studies on Endocrine Disruptors Chemicals. We evaluated and described animal studies, but mainly focused on the in-human ones. The keywords we used were: Endocrine Disruptors Chemicals, Thyroid disruptor chemicals, Industrial chemicals, Thyroid and Polychlorinated Biphenyls, Thyroid and Polybrominated Diphenyl Ethers, Thyroid and Perchlorate, Thyroid and Bisphenol-A and Phthalates, Thyroid and Pesticides and Thyroid and Perfluoroalkyl. We also searched for the latest press releases from the European commission; moreover, we evaluated the potentials in vivo and in vitro models recently developed and used to evaluate the EDC action on thyroid.

## 2. Industrial Chemicals

There are several industrial chemicals recognized to interfere with the hypothalamus pituitary thyroid (HPT) axis. Among them, Polychlorinated Biphenyls (PCBs), Polybrominated Diphenyl Ethers (PBDEs), Perchlorate, Bisphenol-A and phthalates have been extensively studied.

### 2.1. Polychlorinated Biphenyls (PCBs)

PCBs are chemical compounds widely used in pesticide industry before the 70s, when they have been banned; despite several decades, PCBs are still contaminating the environment, being in contact with humans through the food chain [2,9]. In particular, the exposure to PCBs has been linked to cognitive development impairment [10]. In several animal studies, with both rats and monkeys, exposure to PCB demonstrated to reduce the thyroid hormone levels, particularly thyroxin (T_4_) [9]. Few studies have been conducted in humans and the results are not homogenous: some studies found a reduction of thyroid hormones or increase of TSH levels after PCBs exposure, but not all the studies had the same results [9]. In a human study on toxic exposure, among several other toxics and congeners, PCB was measured in maternal milk. The mean levels of total PCB-dioxin toxic equivalent (TEQ) found in milk was 74.86 pg TEQIg fat, the planar-PCB TEQ 19.95 pg TEQ/g fat and the nonplanar-PCB TEQ was 22.75 pg TEQ/g fat [11]. In this study, it has been detected that high concentration of PCB in maternal milk was associated with reduced levels of maternal total triiodothyronine (TT_3_) and total T_4_ (TT_4_) and higher values of thyroid stimulating hormone (TSH) in new-borns [11]. Conflicting results were found when T_4_ and TSH were evaluated in the umbilical cord and PBC in maternal milk, in a standard USA exposure population [12]. The hypothesis behind this action was that PCBs alter thyroid status impacting on deiodinase function [13]. Abdelouahab et al. conducted a large prospective study evaluating the levels of thyroid hormones, PCB and PBDEs in women in the first trimester of pregnancy; at delivery, thyroid hormones were measured in the cord blood. In the cohort, 3 different PCBs congeners were examined: PCB 153, with a maximum concentration detected of 409.92 ng/g lipids, PBC 138, with a maximum concentration detected of 177.63 ng/g lipids and PCB 180, with a maximum concentration detected of 123.91 ng/g lipids. There was a negative correlation between free T_3_ (FT_3_) and PCB levels, and no correlation was found with the hormone levels in the cord blood [14]. A very recent study conducted on a population of electronic waste recycling workers didn’t show any correlation between thyroid hormone and serum concentration of several PCBs (ranging from 204 to 11846 ng/g lipids in men—mean 3173 ng/g lipids—and 136–11,117 ng/g lipids in women, with a mean of 3423 ng/g lipids) or hydroxylated PCB (OH-PCB) levels, ranging from 40.2 to 5233 ng/g lipids in men and 4.24 to 4161 ng/g lipids (mean 1239 ng/g lipids in men and 923 ng/g lipids in women) examined in the population [15]. The potential effect of perinatal exposure to PCBs and hydroxylated PCBs (OH-PCBs) from the background on thyroid hormones in serum and cord blood was assessed in a cohort of 100 mothers and infants. Ten different PCBs (sum of PCBs levels detected ranging from 216.5 to 390.4 ng/g lipids—median 293.4 ng/g lipids) and six OH-PCBs were measured (sum of OH-PCBs ranging from 0.281 to 0.531 ng/g—mean 0.386 ng/g) in the maternal blood, and T_4_, T_4_ sulfate (T_4_S), T_3_, reverse T_3_ (rT_3_) TSH and thyroxin binding globulin (TBG) were measured in the cord blood as well as in serum at three and eighteen months of age. While no correlation was found between PCBs, T_4_, TSH and TBG values in the cord blood, there was a positive correlation between two congeners of the PCBs and the sum of ten PCBs dosed and the T_3_/rT_3_ ratio in the cord blood. One of the hydroxylated congeners was also correlated with serum T_4_ at 3 months of age and with T_4_, T_4_S and T_3_ at eighteen months of age [13]. These findings suggest an effect of PCBs on thyroid hormone metabolism rather than synthesis and secretion.

### 2.2. Polybrominated Diphenyl Ethers (PBDEs)

PBDEs are a group of chemicals produced as flame retardants to delay or prevent potential ignition in fabric and plastic products, paints, electrics and mattresses [9]. The major production was in North America, therefore this region has a very high level of the compounds [10]. The main congeners of PBDEs were penta-BDE, octa-BDE, and deca-BDE; within them, the latter is still in use [10]. The lipophilicity of the congeners, in addition to the fact that they are not chemically bound to the material but simply added, determine an easier absorption and accumulation into several tissue after exposure [2]. Moreover, PBDEs have a chemical structure quite similar to T_4_, dealing with a potential interference with the normal thyroid function [2]. Few data are available and came mainly from animal studies. The first study was done in 1994 by Fowles et al.: in mice treated with both acute and subacute concentration of PBDEs the level of FT_4_ was reduced; in the case of subacute exposure, the reduction was dose-dependent [16]. Other few studies have been conducted in the following decades. In 2010, Lee et al. evaluated the impact of the exposure to decabromodiphenyl ether (BDE-209, the PBDE found in humans at the highest level) in a population of Sprague-Dawley male rats. The thyroid was one of the organs evaluated in the study; total level of T_3_ was reduced while TSH value was increased. Moreover, the exposure to high doses determined histological changes in the thyroid gland, with degenerated or attenuated follicular epithelium [17]. Fewer studies have been conducted in humans. Two different studies evaluated the potential correlation between PBDEs levels and thyroid function in the early 2000s. The first one evaluated the levels of PBDEs in maternal and foetal serum levels together with thyroid hormones in an Indiana population. Despite the higher levels of the compound in both maternal and foetus serum compared to other populations (fetal serum sum of BDE congeners concentration 14–460 ng/g lipids, mean 39 ng/g lipids; maternal serum sum of BDE congeners concentration 15–580 ng/g lipids, mean 37 ng/g lipids), no correlation between the PBDEs and thyroid hormone levels was demonstrated [18]. A different study conducted in a population of workers in an electronic recycling facility. The study examined plasma samples in the cohort at different time-points, beginning with unexposed evaluation to the evaluation after being exposed and assigned to different tasks, such as dismantling, sorting or other work. At the stage of non-exposure, the median level of the sum of BDE was 7.2 pmol/g lw After exposure, and in particular after being assigned to the sorting and dismantling jobs, the median for congeners PBDE 47 (5.7 and 3.7 pmol/g lw respectively), PBDE 28 (2.0 and 0.26 pmol/g lw respectively) and 154 (0.29 and 0.24 pmol/g lw respectively). Despite the changing in the PBDE levels, non-significant, the study failed to demonstrate a relationship between exposure to PBDEs and thyroid hormones; however, the cohort analyzed was very small [19]. Other studies, on the other hand, highlighted a relationship between PBDEs and thyroid hormone levels, with a hyper-thyroidogenic effect of several congeners of the compound. In the HOME study (Health Outcomes and Measures of the Environment), increased T_3_ and T_4_ levels were found associated to the maternal serum levels of two congeners of PBEs (28 and 47, with a median concentration of 1.0 and 19.1 ng/g lipid respectively), in the second trimester of pregnancy; a significant trend was found also between maternal levels of TT_4_ and PBE-47 in the third trimester of pregnancy [20]. These results were in line with the literature of the last decade; in a cohort of one hundred women past their 34th week of pregnancy, Stapleton at al. found a positive association between the congeners BDEs-47, -99, and -100 serum concentration (ranges: BDE-47: <2.0–297.45 ng/g lipids, geometric mean 16.5; BDE-99: <2.0–249.08 ng/g lipids, geometric mean 4.72; BDE-100: <1.2–107.45 ng/g lipids, geometric mean 4.19) and increased levels of FT_4_ and TT_3_, remaining after corrections for different variables [21]. In the same cohort evaluated for PCBs, Abdelouahab et al. found reduced TT_4_ and TT_3_ and increased free fraction for both hormones in relation to some BBDEs levels; the BBDE examined were PBDE 47, 99, 100 and 153, with a maximum concentration detected of 547, 96.1, 82.73 and 85.92 ng/g lipids, respectively. At the time of delivery, a relationship between the PBDEs level and reduced levels of TT_4_, FT_4_ and TSH was noticed [14]. The same study evaluating the effect of PCBs on thyroid function in population of electronic waste recycling workers evaluated the relationship with thyroid hormones and different PBDE. The compounds examined were penta-PBDE, (range 17.1–242 ng/g lipids—mean 104 ng/g lipids—in men; 44.6–853 ng/g lipids—mean 125 ng/g lipids—in women), octa-BDEs (range 8.02–283 ng/g lipids—mean 77.3 ng/g lipids—in men; 4.58–2667 ng/g lipids—mean 287 ng/g lipids—in women), deca-BDEs (range 64.5–1494 ng/g lipids—mean 509 ng/g lipids—in men; 100–34,482 ng/g lipids—mean 1896 ng/g lipids—in women), all congeners together (range 105–1806 ng/g lipids—mean 690 ng/g lipids—in men; 206–35,902 ng/g lipids—mean 2309 ng/g lipids—in women); the study showed a positive association between thyroid hormones and some low brominated congeners from a mixture of BDEs; a negative association was also seen between TSH and some highly brominated BDEs of the mixture used [15]. Zheng et al. studied a cohort of 72 pair mother-foetus in Wenling, China, an area where the exposure to PBDE is due to the electronic waste activity. The cohort was divided in two groups: R_20_, constituted by people who lived in the area for >20 years, and R_3_ group, composed by women who lived in the area for less than 3 years. The median levels of the total PBDEs in the R_20_ group was 19.3 ng/g lw in the serum, 6.84 ng/g lw in the umbilical cord serum, and 2.20 ng/g lw in the placenta; in the R_3_ group, the median concentration of the all PBDEs was 8.13 ng/g lw, 4.47 ng/g lw and 1.06 ng/g lw. A significant difference in the concentration of low brominated PBDEs was found between the mothers’ serum and the cord blood, indicating that the placenta might partially act as a barrier for the passage of the congeners, especially for the high brominated ones. Moreover, one of the congeners (BDE-153) showed a significant correlation with TT_4_ levels in maternal serum [22]. The Deca-BDE and existing products may leak PBDEs into the environment. A study investigated the effect of the Penta-BDE mixture DE-71 on human thyroid cells in vitro. DE71 inhibited differentiated thyroid cell functions in a two phase response manner and a concentration-dependent inhibition of thyroglobulin (Tg) and cAMP production, respectively, as well as expression of mRNA encoding Tg, thyroid peroxidase (TPO) and TSH receptor. This study confirmed an inhibiting effect of PBDEs on thyroid cells [23].

### 2.3. Perchlorate

Perchlorate is a substance used in rocket propellent, airbag manufacture, and fertilizers. It is also a food contact approved substance, and can migrate into food, water and milk [24,25]. Perchlorate acts as an inhibitor of the sodium-iodine symporter (NIS), located on the membrane of thyroid follicular cells and breast cells; the perchlorate binding to NIS impairs thyroid iodine uptake, impacting on the normal functionality of the gland [24]. Different human studies showed inhomogeneous results; the analysis of several data from the U.S. National Health and Examination Survey (NHANES), evaluating subjects from 2001 to 2002, showed a negative association between perchlorate in the urine samples (geometric mean of urine perchlorate concentration in the cohort 2.84 μg/L) and TT_4_ in the face of a positive association with TSH, only in women, especially in women with low urinary iodine concentration (<100 μg/L) [26]. A following analysis of samples from 2001 and 2002 plus samples from 2008 and 2009 evaluated the potential relationship between urinary perchlorate, nitrate, and thiocyanate with serum FT_4_. The overall meta-analysis of the data showed that urinary perchlorate, nitrate and thiocyanate were predictors of FT_4_ level only in non-pregnant women, but the relationship wasn’t found in pregnant women or in men [27]. A large very recent study evaluated 3151 subjects, aged in a range from 12 to 80 years, recorded in the NHANES database from 2009 to 2012. The aim of the study was to evaluate the effect of NIS inhibitors on thyroid function, with particular focus in identifying the sub-population at higher risk for thyroid disruption. The median of the concentration of urinary perchlorate was 3.0 µg/g creatinine, urinary thiocyanate 1.04 mg/g creatinine and urinary nitrate was 40.5 mg/g creatinine. The results of the study showed that the adolescent population is the most sensitive to the action of NIS inhibitors [25]. In a large cohort of hypothyroid/hypothyroxinemic pregnant women in the multicentre Controlled Antenatal Thyroid Screening Study (CATS), a retrospective analysis aimed at evaluating the impact of maternal perchlorate in the first trimester of pregnancy, demonstrated a significant association with reduced Intelligence Quotient in the offsprings. Urine perchlorate was identified in all women (mean 2.58 μg/L). It is worth noticing that the Intelligence Quotient was in the lower 10th percentile in the offspring of mothers with the highest perchlorate levels [28].

### 2.4. Bisphenol-A and Phthalates

Bisphenol-A (BPA) and phthalates are widely used compounds; they are used in several manufactures such as toys, cosmetics, tubes, food packaging, and building appliances. Considering their wide use plus the fact that they are not chemically bound to the material, the exposure of the population is quite diffuse [10,29]. Few animal studies showed that exposure to DBP and DEHP might lead to thyroid disruption, in particular with reduced hormone levels or iodine uptake [30]. In a large cohort (408 subjects) of men referred to the fertility center in the Massachusetts General Hospital between 2000 and mid-2004, urinary concentration of phthalates metabolites and serum FT_3_, FT_4_ and TSH were assessed. The geometric mean of the urine phthalate metabolites found in the whole cohort was: MEP 184 ng/mL, MBP 16.7 ng/mL, MBzP 7.70 ng/mL and MEHP 0.28 ng/mL. The research group found an inverse association between the urinary mono(2-ethylhexyl) phthalate (MEHP) and serum T_3_ levels [30]. Boas et al. evaluated the relationship between urine concentration of six different phthalates and thyroid hormones in a cohort of children. In boys, no association was detected between urinary phthalate metabolites and TT_4_, FT_4_ and TSH; in girls, a significant negative association was found between T_3_ and phthalate metabolites, with some differences according to the phthalate metabolite examined. Taken together the 845 children, a significant negative association was found between urinary phthalate metabolites not corrected for creatinine, TT_3_ and FT_3_. The median concentration of urinary phthalate metabolites was as follows (M/F): MEP 21/21, MBP 130/121, MBzP 17/12, MCiOP 7.2/6.5 μg/L [31]. More recently, in the NHANES 2007–2008 survey the data on urinary samples from 1346 adults and 329 adolescents have been analyzed to evaluate possible associations between phthalate and BP-A exposure and serum thyroid hormone levels. In adults, the creatinine-corrected urinary phthalate metabolite concentrations were as follows: MEHP 2.63 µg/g creatinine, MEHHP 20.6 µg/g creatinine, MEOHP 11.2 µg/g creatinine, MECPP 30.6 µg/g creatinine, MiBP 6.63 µg/g creatinine, MnBP 17.5 µg/g creatinine, MCPP 2.42 µg/g creatinine, BPA 2.03 µg/g creatinine. In adolescents (aged between 12 and 19 years), the values were as follows: MEHP 2.38 µg/g creatinine, MEHHP 23.5 µg/g creatinine, MEOHP 13.31 µg/g creatinine, MECPP 34.72 µg/g creatinine, MiBP 8.01 µg/g creatinine, MnBP 20.65 µg/g creatinine, MCPP 3.01 µg/g creatinine, BPA 1.88 µg/g creatinine. The results showed that urine concentrations of phthalate metabolites were associated with lower T_4_ and T_3_ or higher TSH values, with some differences between males and females. Moreover, urinary bisphenol-A showed a negative relationship with serum TSH levels [32]. Accordingly, an inverse association between urinary concentration of BPA and TSH in pregnant women was recently reported in a case control study [33]. Andrianou et al. carried out a case control study in Cyprus and Romania, to evaluate whether thyroid nodular disease could be associated with BPA and its derivatives, as well as with bisphenol F (BPF). In the cohort of adult women evaluated, although urinary BPA concentrations (urinary BPA concentration levels 2258 ng/L and BPF 465 ng/L) and serum TSH values resulted significantly associated, no relationship was found with the prevalence of thyroid nodular disease [34]. The Hokkaido study, conducted on a cohort of 283 women 23–35 weeks pregnant between July 2002 and October 2005 living in Sapporo and surrounding areas [35]. The concentration of BPA in the cord blood was measured, with a limit of quantification (LOQ) at 0.040 ng/mL. TSH and FT_4_ were obtained from newborns at 3 and 7 days of age; while a negative and sex dependent association was find with sexual hormones, no association was found between blood cord levels of BPA (mean of cord blood BPA 0.051 ng/mL) and thyroid hormones in this cohort [35]. Another study, aiming to evaluate whether a relationship was present between BPA, measured in urine and serum, and iodine levels in subject with nodular goiter (NG) and papillary thyroid carcinoma (PTC) as well as healthy controls, was recently conducted [36]. BPA was detected in all serum samples (4.03 to 13.82 ng/mL) with no differences across the groups; the urinary BPA concentration was higher in the disease groups compared to the healthy control one (142.90 to 1409.90 μg/L, *p* = 0.00 for the NG and *p* < 0.04 for the PTC group), and no difference was found between the NG and the PTC groups. The urinary iodine levels were also higher in the NG and PTC group; a significant correlation between urinary BPA concentration and urine iodine concentration (UIC) was found, suggesting that these two findings are associated with the thyroid pathologies examined [36]. Considering the importance of the BPA exposure in in children and adolescents, Wang et al. evaluated whether an association was detectable between BPA, thyroid volume and nodules, in a population of Chinese children (n 718). Urine BPA iodine and creatinine were measured and thyroid US was done to evaluate thyroid volume and quantify nodules [37]. In 99% of the urinary sample was detected BPA, with concentration of 2.64 μg/g creatinine for boys and 2.35 μg/g creatinine for girls, increasing with age. Fourteen percent of the sample had thyroid nodules; an inverse association was found between urinary BPA concentration and both the thyroid volume and the risk of Thyroid nodules [37]. In the HOME study, urinary and serum concentration of BPA were measured in pregnant women at 16 and 26 weeks of pregnancy, and TSH, T_3_, FT_4_ and T_4_ were measured in serum at 16 weeks of pregnancy and in the cord serum at delivery. The overall mean concentration of urinary BPA 2.2 µg/g Cr; the serum BPA concentration was 2.0 µg/g Cr at 16 weeks, and 2.3 at 26 weeks. No association was found between maternal urinary BPA concentrations and THs measured in cord serum; in girls, lower cord TSH was associated with the 10-fold increased BPA in maternal urine. In maternal and cord serum, no significant association was found between BPA concentration levels at 16 weeks and THs; however, the BPA maternal serum concentration at 26 weeks was associated with lower TSH in girls [38].

The studies reported and a summary of the results is provided in Table 1.

## 3. Pesticides

The association between thyroid dysfunction and pesticides, insecticides, fungicides and fumigants has been widely analysed. Organochlorine (OC) pesticides have a similar structure to T_3_ and T_4_; therefore, they might mimic the activity of thyroid hormones by binding their receptor, so leading to thyroid disruption and dysfunction [39]. Several studies have been conducted to evaluate the potential thyroid disruption after exposure to pesticides. In different animal models Dichlorodiphenyltrichloroethane (DDT) demonstrated its thyroid toxicity, from reducing the capacity to concentrate iodine to histological changes [40]. The cytophysiological changes in the follicular epithelium have been recently studied in rats exposed to low doses of DDT. After exposure to low doses of DDT for 4 weeks, T_4_ levels were reduced in the exposed rats, the size of the follicles was reduced and the epithelial cells of the follicles showed a decrease in length and amounts of microvilli. A reduction in the areas of granular endoplasmic reticulum was also found, and a decreased number of lysosomes detected, when compared to control rats [41]. Six weeks after the exposure started, T_3_ production increased. Structural changes occurred in the follicular cells, indicative of reabsorption processes and thyroglobulin disintegration. After 10 weeks from the beginning of the experiment, there was a reduction in both T_3_ and TSH compared to the control group; the follicle epithelium resulted formed by cells characterized by intense activity as well as other cells unresponsive to TSH stimuli. Long term exposure to low doses of DDT resulted in ultra-structural alterations, impaired regulation of TSH response and switch from a merocrine to a micro-apocrine pattern of secretion [41]. Few studies have been conducted in humans over the last decade, with heterogeneous results [42]. A cross sectional study was carried out on a large population (303 men and 305 women) in a highly contaminated rural area in Brazil, to evaluate potential relationship between 19 different OC pesticides and the levels of thyroid hormones, TSH, anti-TPO antibodies (TPOAb) and Tg [39]. In this study, the prevalence of individuals with positive TPOAb titres as well as with subclinical hyperthyroidism (i.e., reduced serum TSH in the face of normal FT_3_ and FT_4_ values) was higher compared to other regions with different exposure. Moreover, the association between thyroid function and pesticides was differing across gender: in men, TT_3_ values correlated with lower endosulphan 2 (serum median concentration 0.23 ng/mL in men and 0.24 in women), while there was an inverse relationship between T_4_, beta-hexachlorocyclohexane (HCH) (serum median concentration 6.00 ng/mL in men and 6.98 ng/mL in women) and *p*,*p*′-DDT (serum median concentration 3.09 ng/mL in men and 3.20 ng/mL in women). Conversely, T_3_ levels were associated with higher alpha-chlordane (serum median concentration 0.23 ng/mL in men and 0.27 in women), *p*,*p*’-DDT, endosulphan 2 and methoxychlor in women (serum median concentration < limit of detection in men and in women), while T_4_ levels were positively associated with hexachlorobenzene (HCB) (serum median concentration 0.33 ng/mL in men and 0.37 in women), heptachlor (serum median concentration 0.31 ng/mL in men and 0.35 in women) and *p*,*p*’-DDT and *o*,*p*’-DDT (serum median concentration 0.30 ng/mL in men and 0.42 in women) [39]. The prenatal exposure to pesticides has also been evaluated in a cohort of new-born in a region of southern Spain, measuring 17 different OCPs in placentas and TSH in the umbilical cord blood. Within the pesticides analysed, endrin concentration in placenta (geometrical mean of 2.53 ng/g placenta) was associated with increased TSH in the cord blood, and marginally significant association was found with other few pesticides analysed [42]. In another Spanish cohort, Lopez-Espinoza et al. found an association between maternal serum concentrations of 4,4′-dichlorodiphenyldichloroethylene (DDE) measured at 12 weeks of pregnancy and mean (geometrical mean and median resulting 2.0 ng/mL, 1.3 ng/mL and 1.1 ng/mL respectively), increased TSH and reduced FT_4_ levels [43]. Very recently, Hernández-Mariano et al. analyzed serum concentrations of TSH, T_4_ and T_3_ and *p*,*p*′-DDE values in a large cohort of pregnant women, within the 16th week of pregnancy, in a Mexican floriculture area. This study showed a significant positive association between *p*,*p*’-DDE measured (which had a median concentration of 1.2 ng/mL in wet basis and 53.09 ng/g in lipid basis in the subjects where the compound was > the limit of detection) and T_3_ levels, suggesting that even a low dose of exposure might impact on thyroid function [44]. The studies reported and a summary of the results is provided in Table 2.

## 4. Perfluoroalkyl

Perfluoroalkyl substances (PFASs) have been widely used as surface coating in several industrial production settings, from textile and food packaging to cosmetic and photography. The correlation between different PFASs with thyroid hormones have been evaluated in several human studies, showing different trend depending on the sex and the age of the cohort [45]. The CHIrP (Chemicals, Health and Pregnancy) study, conducted in Canada among a population of 152 euthyroid pregnant women, assessed the levels of different PFASs (perfluorohexanesulfonate (PFHxS) median concentration detected 1.0 ng/mL, perfluorononanoate (PFNA) median concentration detected 0.6 ng/mL, perfluorooctanoate (PFOA) median concentration detected 1.7 ng/mL and perfluorooctanesulfonate (PFOS) median concentration detected 4.8 ng/mL) in the maternal serum together with thyroid hormones, TSH and TPOAb. A positive association between TSH and PFASs was found in women with positive TPOAb titres, along with a weak association with reduced FT_4_; exposure to PFASs might exacerbate the thyroid hormone alteration seen during pregnancy, impacting on health foetal development [46]. The Northern Norway Mother-and-Child contaminant Cohort Study (MISA) investigated the potential association between thyroid hormones, thyroid binding protein, TPOAb and different PFASs (PFOS, perfluorodecanoate (PFDA) and perfluoroundecanoate (PFUnDA)) in three samples of maternal blood collected in the second trimester of pregnancy and 3 days and 6 weeks after delivery. In women in the highest quartile of PFOS (range 11.1–35.9 mg/mL) there was a positive association between the compounds and TSH levels, while women in the highest quartile of PFDA (range 0.31–2.34 ng/mL) and PFUnDA (range 0.4–0.96 ng/mL) had reduced levels of TT_3_ and FT_3_ [47]. A systematic review of the available epidemiological studies has been recently published: studies focusing on the relationship between TSH, T_3_, T_4_ and different PFAS (in particular PFHxS, perfluorooctanoic acid, PFOS or perfluorononanoic acid) in pregnant women or young children were selected. Some interesting results have been highlighted such as a positive association between PFHxS, PFOS and TSH levels in maternal blood, as well as between PFNA and TSH in boys older than 11 years, but the heterogeneity of the studies included in the review needs to be accounted for [48].

The studies reported and a summary of the results is provided in Table 3.

## 5. Potential New Models to Evaluate EDCs

The importance of the impact of thyroid disrupting chemical on the hormonal homeostasis raises the need of adequate essays, both in vivo and in vitro, to study the physiopathology of the damage and standardize the research metodology. Considering that water is a contaminated environment and route of distribution for pollutant, the levels accumulated in fishes and amphibians could be considered efficient indicator [49]. Zebrafish in particular is known to be a good model, and has been already routinely used [50]. Considering that the HPT axis is highly conserved within the species, several studies have been conducted with zebrafish [49]. Ji et al. measured the expression of an enhanced green fluorescent protein (EGFP), controlled by the TSHβ [49]. After stimulation with several goitrogen compounds, the expression of EGFP was consistent with the endogenous TSHβ mRNA expression, confirming the potential validity of the model [49]. Terrien et al. conducted a molecular study to evaluate the effect of BPA in a transgenic model of zebrafish (TH/bZIP). They measured the response to TH agonists, such as triiodothyroiacetic acid, and antagonists such as NH_3_ and sodium perchlorate (NaClO4). Moreover, the expression of genes in the thyroid axis after exposure to T_3_ or BPA was measured [50]. The exposure to NH_3_ and NaClO_4_ showed a reduction in fluorescence, therefore in thyroid function. The exposure to BDA on its own didn’t determine any reduction in fluorescence, but when tested together with T_3_ it showed a reduction in T_3_-induced fluorescence, indicating an interference of BDA with thyroid functioning [50]. The same association determined a modified expression of few regulatory genes [50]. *Xenopus laevis* is another animal model used to evaluate morphological developmental problem due to different signal alterations. Very recently, Mendelin et al. studied the potential action of thyroid disruptors tetrabrominated diphenyl ether 47 (BDE-47) and tetrabromobisphenol-A (TBBPA) in a tadpole model from a transgenic line of *Xenopus Laevis* [51]. BDE-47 didn’t show a direct thyroid impact, but a rather general toxicity, while TBBPA might have a disrupting action. Overall, the transgenic model resulted to be an efficient essay [51]. In vitro studies have also been conducted to evaluate the potential pathophysiology of the endocrine disruption [52]. Nine different model compounds (thyroid agonists or antagonists) and three different water extracts were evaluated using six different in vitro essays targeting different thyroid function indicators (synthesis and transport of THs, and TH receptor mediated effect) plus one in vivo model [52]. Some promising results have been obtained, particularly using the essays targeting transthyretin (TTR) displacement and TPO inhibition; the in vivo model showed promising results as well [52].

## 6. Conclusions

Several classes or endocrine disrupting chemicals have been studied over the past decades, and few statements have underlined the relevance of environmental exposure on the whole endocrine system. Unfortunately, not so many studies have been carried out on that topic. Moreover, the differences in the population and areas selection, the exposure rate, the time of exposure of the cohort examined and the age range represent a limitation for an overall conclusion. It is certain that industrial chemicals are impacting on the endocrine system in many ways and in different steps of the specific axis. Since a correct thyroid function is widely recognised to be crucial for several biological functions including those of the cardiovascular, osteo-muscular, cognitive and immune systems, larger studies and more homogeneous and reliable data should be addressed as a priority. The data summarized in the current review are in line with the considerations of the European Union Commissioner for Health and Food Safety in occasion of the accomplishment of the scientific criteria to identify endocrine disruptors in the field of plant protection products voted in July 2017 by the member States representatives. Once implemented, the recommendations of the European Commission will ensure that any active substance used in pesticides which is identified as an endocrine disruptor for people or animals can be assessed and withdrawn from the market, a fundamental step towards greater protection of citizens from harmful substances.

## Figures and Tables

**Table 1:** Major studies on industrial chemicals and main results obtained.

Compound	Aim of the Study	Result	References
Polychlorinated Biphenyls	Evaluated the maternal exposure to 26 PCBs (and dioxin) in maternal plasma and umbilical cord plasma during the last month of pregnancy, in umbilical cord plasma and in human milk, and the relationship with thyroid hormones.	↑PCB levels in human milk correlated significantly with ↓plasma levels of maternalTT_3_ and TT_4_. ↑plasma levels of TSH in the babies in the 2nd week and 3rd month. Infants exposed to ↑toxic doses had ↓plasma FT_4_ and TT_4_ in the 2nd week after birth.	[11]
	Evaluated the correlation between several PCBs in maternal blood during pregnancy and T_4_, T_4_S, T_3_, rT_3_, TSH and TBG levels in cord blood/serum at three- and 18-month-old babies.	Positive correlation between 3 PCBs and T_3_ (cord serum). Negative correlation between 4 PCBs and rT_3_ (cord serum). After correction, 2 PCBs and the sum of the 10 PCBs showed positive correlation with the cord serum T_3_/rT_3_ ratio. No correlations between PCBs and T4, TSH and TBG in cord blood. Positive correlation between 4-OH-PCB-107 and T_4_ at 3 months and T_4_, T_4_S and T_3_ at 18 months.	[13]
	Evaluated the associations between maternal blood levels of 3 PCBs congeners and thyroid hormones in maternal and umbilical-cord blood in pregnant women in the first trimester of pregnancy. Thyroid hormone levels also assessed at delivery and in cord blood in 260 subjects.	At delivery, negative associations between maternal FT_3_ and PCBs.	[14]
	Analysed the relationship between serum concentrations of PCBs, levels of thyroid hormones and the mRNA levels of seven TH-regulated genes in peripheral blood leukocytes of e-waste recycling workers.	No associations of TH and PCBs. TH-regulated gene expression was associated with some PCBs and hydroxylated PCB congeners.	[15]
Polybrominated Diphenyl Ethers	Evaluated the associations between levels in maternal blood of PBDEs and levels of thyroid hormones in maternal and umbilical-cord blood in a 380 pregnant women in the 1st trimester of pregnancy. Thyroid hormone levels also assessed at delivery and in cord blood in 260 subjects.	Before 20 weeks of pregnancy, inverse association between maternal PBDEs and total T3 and total T4 and a direct association with free T3 and free T4 were observed. At delivery, negative associations between maternal T T_4_, FT_3_, cord-blood FT_4_, and PBDEs.	[14]
	Analyzed the relationship between serum concentrations of PBDEs, thyroid hormones TH and mRNA levels of seven TH-regulated genes in peripheral blood leukocytes of e-waste recycling workers.	↑T4 and T3 levels associated with some lower-brominated BDEs. Negative association between highly brominated PBDE and TSH levels. The expression of most target genes was suppressed by PBDEs (mostly highly brominated congeners).	[15]
	Correlation between levels of PBDEs in maternal and foetal serum with thyroid hormones in an Indiana population.	No correlation between the PBDEs and thyroid hormone levels.	[18]
	Relationship between PBDEs congeners exposure and thyroid hormones in a population electronic recycling facility workers.	No relationship between exposure to PBDEs and thyroid hormones (small cohort).	[19]
	Relationship between maternal PBDE levels and thyroid hormone levels in maternal and cord sera.	↑T_3_ and T_4_ associated with levels of PBE-28 -47 in 2nd trimester of pregnancy. Significant trend between maternal levels of TT_4_ and PBE7 in the 3rd trimester. No association between maternal PBDE levels and thyroid hormones levels in cord serum.	[20]
	Measured PBDEs and metabolites women in late pregnancy phases. Further objective was the potential association between PBDEs and maternal thyroid hormones.	Positive association between BDEs-47, -99, and -10 and increased levels of FT4 and TT3.	[21]
	Quantified the partitioning of selected PBDEs from mother to foetus and evaluate the effect of PBDE exposure on maternal THs levels.	Significant difference between mother’s serum levels of low brominated PBDEs and the cord blood. Significant correlation between one PBDE and maternal serum T_4_.	[22]
Perchlorate	Evaluated the effect of NIS inhibitors on the thyroid function, and identify the sub-population at higher risk for thyroid disruption; 3151 subjects, 12–80 years.	Adolescents are the most sensitive to the action of NIS inhibitors.	[25]
	Evaluated the relationship between urinary levels of perchlorate and serum levels of TSH and T_4_ in a population from the National Health and Nutrition Examination Survey (2001–2002).	Negative association between perchlorate in the urine samples and TT_4_, and a positive association with TSH, only in women, especially in women with low urinary iodine concentration.	[26]
	Evaluated the association between urinary perchlorate and serum FT_4_ in individuals with ↓urinary iodine levels and pregnant women	Urinary perchlorate is predictor of FT_4_ level only in non-pregnant women.	[27]
	Evaluated the impact of maternal perchlorate in the first trimester of pregnancy, in hypothyroid/hypothyroxinemic pregnant women.	Significant association with reduced Intelligence Quotient in the offsprings, in the lower 10th percentile in the offspring of mothers with the highest perchlorate levels.	[28]
Bisphenol-A phthalates	Evaluated relationship between urinary concentration of phthalates metabolites and FT_3_, FT_4_ and TSH.	Inverse association between the urinary mono(2-ethylhexyl) phthalate levels and serum T_3_ levels.	[30]
	Evaluated the relationship between urine concentration of different phthalates and thyroid hormones in children.	In boys, no association was found. In girls, significant negative association between T_3_ and phthalate metabolites. All cohort: significant negative association between phthalate metabolites and TT_3_ and FT_3_.	[31]
	Evaluated the association between phthalate and BP-A exposure and thyroid hormone levels in the serum.	Association between phthalate metabolites concentration in the urine samples with ↓T_4_ or T_3_ or ↑TSH. Negative association between urinary bisphenol-A and TSH.	[32]
	Evaluated the association between urinary BPA concentrations and plasma thyroid hormone during pregnancy.	Inverse association between urinary concentration of BPA and TSH in pregnant women.	[33]
	Evaluated the association between thyroid nodular disease and BP-A and -F.	In the cohort of adult women evaluated, urinary concentration of BPA and serum TSH value were significantly positively associated, but no association was found with the nodular disease.	[34]
	Evaluated the association between BPA in cord blood and TSH and FT_4_ at 3 and 7 days of age.	No association between BPA levels and TSH and FT_4_ was found in this cohort.	[35]
	Evaluation the relationship between BPA concentration in urine and serum and urinary iodine levels in subject with NG and PTC, and HC.	UBC were higher in the NG and PTC groups compared with HC. UIC were higher in the NG and PTC groups compared with HC. Significant correlation between UBC and UIC in the groups.	[36]
	Evaluated the association between urinary BPA thyroid volumes and thyroid nodules in a population of 718 Chinese children (aged 9–11 years).	99.9% of urinary samples showed presence of BPA. 14% of children had thyroid nodules. Inverse association was found between urinary BPA concentration and both the thyroid volume and the risk of Thyroid nodules.	[37]
	Evaluate whether BPA concentration in urine samples of women at 16 and 26 weeks of pregnancy were associated with THs in maternal or cord serum. Eventual differences between boys and girls were evaluated.	No association between maternal urinary BPA concentrations and THs in cord serum; ↓cord TSH in girls was associated with maternal urine levels increased 10 folds. No association was found between maternal and cord serum BPA at 16 weeks and THs. Association was found between BPA maternal serum concentration at 26 weeks and ↓TSH in girls.	[38]

PCBs: Polychlorinated Biphenyls; TT_3_: total triiodothyronine; TT_4_: total thyroxin; T_4_: thyroxin; T_4_S: thyroxin sulfate: T_3_: triiodothyronine; rT_3_: reverse triiodothyronine; TSH: thyroid stimulating hormone; TBG: thyroxine-binding globulin; FT_4_: free thyroxin; OH-PCB: hydroxylated—Polychlorinated Biphenyls; FT_3_: free triiodothyronine; TH: thyroid hormones; PBDE: Polybrominated Diphenyl Ethers; NIS: sodium-iodine symporter; BP-A: Bisphenol-A; NG: nodular goiter; PTC: papillary thyroid carcinoma; HC: healthy controls; UBC: Urinary BPA concentrations; UIC: urinary iodine concentrations.

**Table 2:** Major studies on pesticides and main results obtained.

Compound	Aim of the Study	Results	Ref.
Pesticides	Evaluated the relationship between 19 different OC pesticides and thyroid hormones, TSH, TPOAb and thyroglobulin (cohort of 303 men and 305 women).	In men, correlation between endosulphan 2 and TT3, inverse correlation between T4 and beta-hexachlorocyclohexane and *p*,*p*′-DDT. In women, association of T3 levels and ↑ alpha-chlordane, DDT, endosulphan 2 and methoxychlor; T4 levels positively associated with HCB, heptachlor, DDT	[39]
	Evaluated relationship between OCPs levels in placenta and TSH in umbilical cord blood.	Endrin was associated with ↑TSH in the cord blood.	[42]
	Evaluated the association between thyroid hormone levels and 4,4′-DDE concentrations in pregnant women	Association found between maternal serum concentration of 4.40-DDE and ↑TSH and ↓FT4	[43]
	Evaluate the effect of exposure to *p*,*p*′-DDE during the first half of pregnancy in thyroid profile	Significant positive association between *p*,*p*′-DDE and T3 levels.	[44]

TT_3_: total triiodothyronine; T_4_: thyroxin; T_3_: triiodothyronine; TSH: thyroid stimulating hormone; OC: Organochlorine; TPOAb: anti thyroperoxidase antibodies; DDT: Dichlorodiphenyltrichloroethane; HCB: hexachlorobenzene; DDE: dichloro diphenyldichloroethylene.

**Table 3:** Major studies on perfluoroalkyl and main results obtained.

Compound	Aim of the Study	Results	Ref.
Perfluoroalkyl	Evaluate the levels of different PFAS, TSH and TPOAb in the maternal serum of euthyroid pregnant women.	Positive association between TSH and PFASs in TPOAb positive women, plus weak association with ↓FT_4_.	[46]
	Evaluate the potential association between thyroid hormones, thyroid binding proteins, TPOAb and different PFASs in three samples of maternal blood in the second trimester of pregnancy and 3 days and 6 weeks after delivery.	Positive association between the compound and TSH levels in women in the highest quartile of PFOS. Women in the highest quartile of PFDA and PFUnDA had reduced ↓ of TT_3_ and FT_3_.	[47]
	Evaluate the epidemiological studies focussing on the relationship between TSH, T_3_, T_4_ and different PFAS in pregnant women or young children.	Positive association between PFHxS and PFOS and TSH levels in maternal blood, Positive association between PFNA and TSH in boys older than 11 year of age.	[48]

TT_3_: total triiodothyronine; T_4_: thyroxin; T_3_: triiodothyronine; TSH: thyroid stimulating hormone; FT_4_: free thyroxin; FT_3_: free triiodothyronine; TPOAb: anti thyroperoxidase antibodies; PFAS: perfluoroalkyl substances PFOS: perfluorooctanesulfonate; PFHxS: perfluorohexanesulfonate; PFDA: perfluorodecanoate; PFUnDA: perfluoroundecanoic acid.
